# Matrix metalloproteinase-7 induces E-cadherin cleavage in acid-exposed primary human pharyngeal epithelial cells via the ROS/ERK/c-Jun pathway

**DOI:** 10.1007/s00109-021-02166-z

**Published:** 2022-01-01

**Authors:** Nu-Ri Im, Byoungjae Kim, Kwang-Yoon Jung, Seung-Kuk Baek

**Affiliations:** 1 Department of Otorhinolaryngology-Head and Neck Surgery, College of Medicine, Anam Hospital, Korea University, 73, Inchon-ro, Seongbuk-gu, Seoul, South Korea; 2 College of Medicine, Neuroscience Research Institute, Korea University, Seoul, Republic of Korea

**Keywords:** Laryngopharyngeal reflux, Matrix metalloproteinase-7 (MMP-7), Reactive oxygen species (ROS), Mitogen-activated protein kinases (MAPK), Transcription factor

## Abstract

**Abstract:**

Laryngopharyngeal reflux disease (LPRD) is caused by pharyngeal mucosal damage due to the reflux of gastric contents, including acid, pepsin, and bile juice. Our previous study has demonstrated that LPRD is associated with the cleavage of E-cadherin, which is facilitated by the acid-activated matrix metalloproteinase-7 (MMP-7); however, the mechanism by which the acid activates MMP-7 remains unclear. The purpose of this study was to investigate the mechanism by which MMP-7 is activated in the pharyngeal epithelial cells that are exposed to acid. The levels of reactive oxygen species (ROS) were measured in the epithelial cells exposed to acid. To investigate the signaling mechanism of ROS in the expression of MMP-7, the mechanism of action of the mitogen-activated protein kinase was examined. The expression of various signaling factors was determined, according to the presence or absence of each inhibitor in the acid-exposed pharyngeal epithelial cells. To identify changes in the cleavage of E-cadherin, the integrity of the mucosal membrane was assessed using a transepithelial permeability test. We found that acid exposure increased the levels of ROS, phosphorylated-extracellular signal-regulated kinase (p-ERK) 1/2, and phosphorylated-c-Jun (p–c-Jun) in pharyngeal epithelial cells. The ROS inhibitor reduced the expression of p-ERK and MMP-7, while the ERK inhibitor reduced the expression of p–c-Jun and MMP-7. Moreover, the c-Jun inhibitor reduced the expression of MMP-7 and blocked the degradation of E-cadherin. In addition, decrease in the levels of immunostained E-cadherin and increase in transepithelial permeability after acid exposure were collectively alleviated by the inhibitors of ROS, ERK, and c-Jun. The degradation of E-cadherin that occurs after human mucosal cells are exposed to acid appears to be caused by an increase in the expression of MMP-7 via the ROS/ERK/c-Jun pathway, which is thought to be an important mechanism associated with the development of LPRD.

**Key messages:**

• ROS is triggered when reflux occurs.

• ROS regulates the transcription factor c-Jun via the ERK pathway.

• The increase in MMP-7 that induces LPRD is induced via the ROS/ERK/c-Jun pathway.

• This study revealed for the first time the expression mechanism of MMP-7, which is one of the causes of LPRD.

**Supplementary Information:**

The online version contains supplementary material available at 10.1007/s00109-021-02166-z.

## Introduction

Laryngopharyngeal reflux disease (LPRD) is caused by the reflux of gastric contents, including acid, pepsin, and bile juice, which causes damage to the larynx and pharynx [1]. Notably, several previous studies have reported that reactive oxygen species (ROSs) play a key role in the development of LPRD [2]. As the previous studies on esophagitis caused by ROS [3–5], ROS may cause pharynx and larynx tissue damage, and the use of antioxidants may reduce the tissue damage caused by ROS.

Oxidative stress is a phenomenon caused by an imbalance in the biological systems that detoxify reactive products, such as ROS, in cells and tissues. Oxidative stress has been associated with the development of several inflammatory diseases, such as cancer, cardiovascular disease, airway disease, and rheumatoid arthritis [6–9]. In these diseases, ROSs are associated with tissue remodeling, cellular injury, and apoptosis. A recent study has demonstrated that ROSs play important roles as signaling molecules that regulate many genes, and they can control the expression and activation of *MMP* genes [10]. Matrix metalloproteinases (MMPs) are enzymes involved in tissue remodeling and degradation of the extracellular matrix. Among these MMPs, matrix metalloproteinase-7 (MMP-7) is expressed by exocrine and mucosal epithelial cells in various tissues and plays an important role in the remodeling of cell surface molecules, such as the epidermal growth factor receptor, heparin-binding epidermal growth factor, Fas ligand, and E-cadherin [11–15]. In addition, our previous study has demonstrated that the expression of MMP-7 increases in pharyngeal epithelial cells exposed to acid and that the mucosal adhesion molecule, E-cadherin, is degraded by MMP-7 [16]. Therefore, the aim of the present study is to investigate ROS-related mitogen-activated protein kinase (MAPK) to find out through what process the increase in MMP-7 expression caused by acid exposure occurred.

Based on the association between ROS and MMP-7 demonstrated in previous studies, it is important to identify the sub-mechanisms linking them in a LPRD model. Thus, in the present study, we investigated the mechanisms of action of various MAPKs that are associated with the ROS/MMP-7/E-cadherin pathway in human pharyngeal epithelial cells exposed to acid.

## Materials and methods

### Tissue preparation

Normal pharyngeal mucosa was harvested from the posterior tonsillar pillar samples of 10 subjects who underwent tonsillectomy at the Department of Otorhinolaryngology-Head and Surgery, Korea University Hospital due to tonsillar hypertrophy and sleep problems (seven men and three women: age range, 19–47 years). The subjects exhibited no signs of acute inflammation of the pharynx, had no history of allergy and smoking, and were not undergoing drug treatment at the time of the study. The study protocols and experimental design parameters were reviewed and approved by the Institutional Review Board of Korea University Hospital (IRB No. ED15303). Informed consent was obtained from all participants, and all methods were performed in accordance with the relevant guidelines and regulations.

### Cell culture

Human pharyngeal mucosal samples were incubated in 1 mg/ml dispase in Dulbecco’s modified Eagle’s medium/F12 (DMEM/F12) for 1 h at 37 °C in 5% CO_2_. The pharyngeal epithelial cells were then freed by curettage and collected into a 15-ml conical tube. After centrifugation, the cells were rinsed three times with DMEM/F12 and cultured in serum-free bronchial epithelial growth medium (BEBM, Lonza, Walkersville, MD, USA) [17]. On reaching approximately 70% confluency, the cells were detached with 0.25% trypsin EDTA, washed in DMEM/F12, and resuspended in BEBM kit media in 12-well culture plates (SPL, Seoul, Korea) at approximately 1 × 10^5^ cells/well. Passage 2 of pharyngeal epithelial cells was used in all the experiments. Routine cultures were maintained in a 5% CO_2_ incubator at 37 °C, and the media was changed every 3 days. Cell morphology was examined using an Olympus CKX41-A32PHP microscope (Olympus, Tokyo, Japan) [16].

### Acid exposure and inhibitor treatment

To mimic acid reflux, confluent pharyngeal epithelial cells were treated with HCl at pH 4.0 for 1 min and 5 min. After treatment, the cells were incubated with non-acidic BEBM at 37 °C in 5% CO_2_ for 24 h, washed twice with phosphate-buffered saline (PBS), and used for experiments.

For the inhibition study, epithelial cells were pretreated with *N*-acetyl cysteine (NAC; ROS inhibitor, Sigma-Aldrich, St. Louis, MO, USA), 10 μM of U0126 (ERK1/2 inhibitor; Cell Signaling Technology [CST], MA, USA), or SP600125 (c-Jun inhibitor; Sigma-Aldrich, St. Louis, MO, USA) and incubated for 30 min at 37 °C in 5% CO_2_. After treatment with HCl as described above, the cells were incubated for 24 h. The control cells were treated with non-acidic BEBM for the same period.

### RT-qPCR

Gene expression in the epithelial cells was assessed using RT-qPCR. Total RNA was extracted from approximately 5 × 10^5^ cells using TRIzol reagent (Qiagen, Germantown, MD, USA) and RNase-free DNase I (Qiagen). RNA (1 µg) was reverse transcribed to cDNA using amfiRivert cDNA Synthesis Platinum Master Mix (GenDEPOT, Gyeonggi-do, Korea). The prepared cDNA was amplified and quantified using the SYBR Green Master Mix (Qiagen) with the following primers: glyceraldehyde-3-phosphate dehydrogenase, forward 5′-GAG TCA ACG GAT TTG GTC GT-3′ and reverse 5′-TTG ATT TTG GAG GGA TCT CG-3′; MMP-7, forward 5′-TGA GCT ACA GTG GGA ACA GG-3′ and reverse 5′-TCA TCG AAG TGA GCA TCT CC-3′; and CDH1 for E-cadherin, forward 5′-TGC TCT TGC TGT TTC GG-3′ and reverse 5′-TGC CCC ATT CGT TCA AGT AG-3′. PCR was performed using a real-time thermal cycler system (TP800/TP860; Takara, Kusatsu, Shiga, Japan) with 40 cycles of a 2-step reaction consisting of denaturation at 95 °C for 15 s, followed by annealing/extension at 60 °C for 45 s. The data were analyzed using the △*Ct* method.

### Western blotting

For the Western blot analysis, we mixed 10 µg of protein from each sample with 5 × Laemmli buffer and 5% *β*-mercaptoethanol and boiled it for 10 min, and the supernatant of each well was concentrated to equal volume using Centricon (3 kDa cut-off; Merck Millipore, Billerica, MA, USA) at 3000 × g for 40 min at 4 °C. The cell and supernatant extracts were separated using sodium dodecyl sulfate–polyacrylamide gel electrophoresis and transferred to nitrocellulose membranes. The membranes were then incubated overnight at 4 °C with antibodies against E-cadherin (1:1000; Santa Cruz Biotechnology, Santa Cruz, CA, USA), MMP-7 (1:1000; Sigma-Aldrich), p38 (1:1000; CST), p-p 38 (1:1000; CST), ERK (1:1000; CST), p-ERK (1:1000; CST), JNK (1:1000; CST), phosphorylated-JNK (p-JNK; 1:1000; CST), c-Jun (1:1000; CST), p–c-Jun (1:1000; CST), nuclear factor-κB (NF-κB; 1:1000; CST), phosphorylated-NF-κB (p-NF-κB; ser 276; 1:1000; CST), and p-NF-κB (ser 536; 1:1000; CST) or anti-β-actin antibody (1:2000; Santa Cruz Biotechnology) for loading the control in the blocking solution. Next, the membranes were incubated with the appropriate anti-rabbit (1:1000; Santa Cruz Biotechnology) or anti-mouse antibody (1:1000; Santa Cruz Biotechnology) in the blocking solution. The blots were visualized using the Chemiluminescence Kit (Santa Cruz Biotechnology), and images were captured with ChemiDoc (Bio-Rad Laboratories, Hercules, CA, USA).

To evaluate the expression of E-cadherin, it is important to preserve the cell–cell junctional adhesions. Therefore, human pharyngeal epithelial cells were collected by scraping the cells to maintain their cell–cell interaction, and the difference of E-cadherin expression was semiquantitatively estimated based on the β-actin expression in each sample. And all other samples were determined according to bicinchonic acid (BCA) protein assay, using bovine serum albumin as a standard, and equal amounts of protein were loaded on the gel.

### Flow cytometry–based measurement of ROS levels

ROS levels in the epithelial cells exposed to acid were evaluated by flow cytometry using a 2′,7′-dichlorofluorescein diacetate assay. Acid-treated cells were scraped and resuspended in ice-cold PBS containing 5% bovine serum albumin. For performing flow cytometry analysis, the cells were stained using an intracellular staining protocol with a fixation/permeabilization buffer solution (BD Biosciences, USA) for 30 min at 4 °C and then incubated with 10 μM of 2′,7′-dichlorofluorescein diacetate (Sigma-Aldrich) in the dark for 30 min. The analysis was performed using FACS Canto II (BD Biosciences).

### Immunocytochemistry analysis

Cells were cultured on cytoslides (Marienfeld-Superior, Lauda-Königshofen, Germany) and treated with acid or inhibitor as mentioned above. Cells were fixed with 4% glutaraldehyde for 30 min and blocked for 1 h at room temperature with goat serum (Vector Laboratories, Burlingame, CA, USA). The cells were then incubated with rabbit polyclonal antibody against E-cadherin (1:50, Santa Cruz) overnight at 4 °C. During the next day, the cells were treated with biotinylated anti-rabbit IgG (H + L) secondary antibody in PBS (1:400) for 60 min at room temperature. After washing the cytoslides with PBS, antigen–antibody complexes were detected using an avidin–biotin complex detection system (Vectastain ABC Kit, Vector Laboratories). The cytoslides were stained using the DAB Substrate kit (Vector Laboratories), rinsed in water, briefly counterstained with Mayer’s hematoxylin, and washed again in water. After mounting on glass slides, the cytoslides were examined using an Olympus BX51 microscope. Pictures were captured and controlled using an Olympus DP72 and DP2-BSW (Olympus).

Immunostaining of E-cadherin was evaluated in five microscopic fields (× 200) of three different samples. To quantify the E-cadherin expression, the number of E-cadherin-stained cells throughout the cell membrane was counted. Then, the semiquantitative score was calculated as the percent of the number of the stained cells per total number of cells in each microscopic field. These results were evaluated by three independent researchers.

### Transepithelial permeability analysis

Human pharyngeal epithelial cells were seeded at a density of 1 × 10^5^ cells/cm^2^ on 12-transwell culture plates with 0.4-μm polyester filters (SPL). The transepithelial permeability test was performed 24 h after acid exposure with/without inhibitor treatment as mentioned above. Fifty microliters of 100 μM rhodamine B isothiocyanate (RITC)-labeled Dextran 70S (Sigma) was added to the top chamber of the transwell plates. For the next 3 h, 50 μl of the media samples was collected from the bottom compartments at 30-min intervals and analyzed on a SpectraMax M2^e^ plate reader (Molecular Devices) with an excitation wavelength of 530 nm and emission of 590 nm (SOFTMAX PRO v5 software, Molecular Devices) [18].

### Statistics analysis

The statistical results are expressed as the mean ± standard deviation (SD) measured after each experiment (*N*). The data were based on one-way analysis of variance, and a *P*-value of less than 0.05 was considered statistically significant.

## Results

### ROS and MMP-7 levels are upregulated in human pharyngeal epithelial cells following acid exposure

**Fig. 1:**
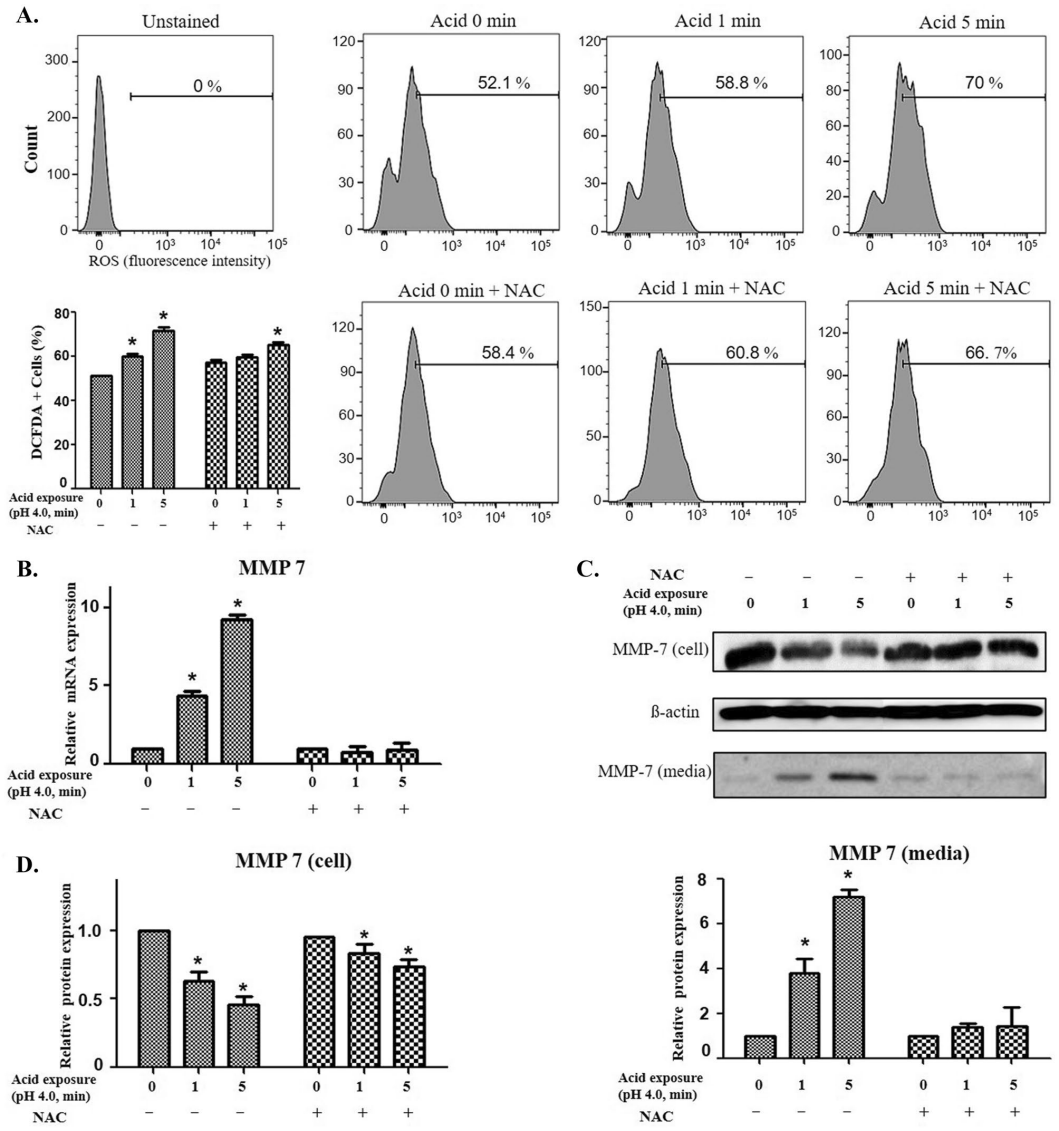
Reactive oxygen species (ROS) and matrix metalloproteinase-7 (MMP-7) levels in human pharyngeal mucosal epithelial cells exposed to acidic media. **A** ROS levels in the epithelial cells exposed to acid increased in proportion to the acid exposure time and significant reduced by treating NAC. Statistical graph according to ROS levels (*p < 0.05). **B** Increase in transcriptional expression of MMP-7 in pharyngeal epithelial cells exposed to acid was reduced after treatment with *N*-acetyl cysteine (NAC). **C** Increased MMP-7 protein in culture media after exposure to acid was significantly decreased after treatment with NAC. **D** The relative expression of MMP-7 in cells based on β-actin levels and the relative expression of MMP-7 in the acid-exposed medium compared to the medium without acid exposure

To determine the signaling mechanism underlying MMP-7 activation, the expression levels of ROS, one of the major regulators of MMPs, were investigated in human pharyngeal epithelial cells exposed to an acidic environment. The generation of ROS increased in proportion to the acid exposure time in human pharyngeal epithelial cells, which was later reduced following treatment with NAC (Fig. 1A). In addition, the increased transcriptional expression (Fig. 1B) and secretion of MMP-7 (Fig. 1C and D) caused by acid treatment were significantly decreased after treatment with NAC. These results indicate that the expression of MMP-7 in pharyngeal epithelial cells exposed to an acidic environment may be regulated by ROS.

### Phosphorylation of ERK is regulated by ROS in pharyngeal epithelial cells

**Fig. 2:**
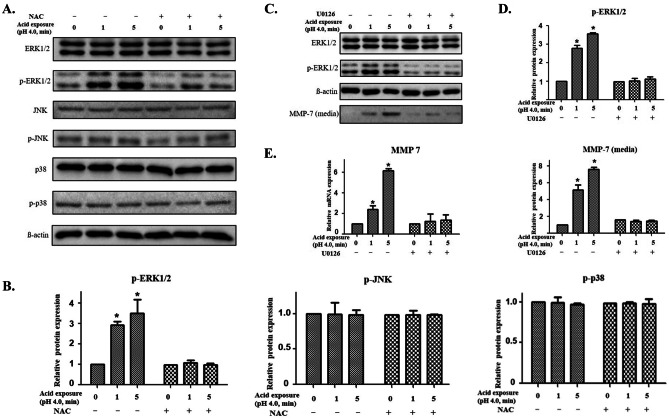
Regulation of the mitogen-activated protein kinase (MAPK) pathway after acid treatment. **A** The phosphorylation of extracellular signal-regulated kinase (ERK1/2) was increased dependent on the acid exposure time and was reduced after the treatment of NAC. **B** The relative expressions of phosphorylated kinases based on ß-actin level. **C** Inhibition of the ERK pathway by U0126 inhibited the phosphorylation of ERK1/2, and it reduced MMP-7 increased in culture media after acid treatment. **D** The relative expression of phosphorylated ERK1/2 in the cell based on β-actin level and the relative expression of MMP-7 in the acid-exposed medium compared to the medium without acid exposure. **E** The transcriptional expression of MMP-7 was inhibited by U0126

The phosphorylation of MAPK including ERK 1/2, JNK, and p38 results in an activation of its kinase activity and leads to phosphorylation of its many downstream targets. When the pharyngeal epithelial cells were treated with acid for 1 min and 5 min, the phosphorylation of ERK1/2 increased depending on the acid exposure time, and it showed an apparent decrease following NAC treatment. However, the phosphorylation of JNK and p38 was not affected by acid or NAC treatment (Fig. 2A and B). In addition, measurement of the inhibitory effect of U0126 on ERKs revealed that the phosphorylation of ERK1/2 was significantly inhibited, while the secretion of MMP-7, increased by acid exposure, was not elicited (Fig. 2C and D). Similarly, the expression of MMP-7 was significantly reduced at the transcriptional level when U0126 was used (Fig. 2E). These results suggest that increased MMP-7 expression induced by ROS may occur through the ERK signaling pathway.

### ERK/c-Jun signaling regulates the expression of MMP-7 and the degradation of E-cadherin

**Fig. 3:**
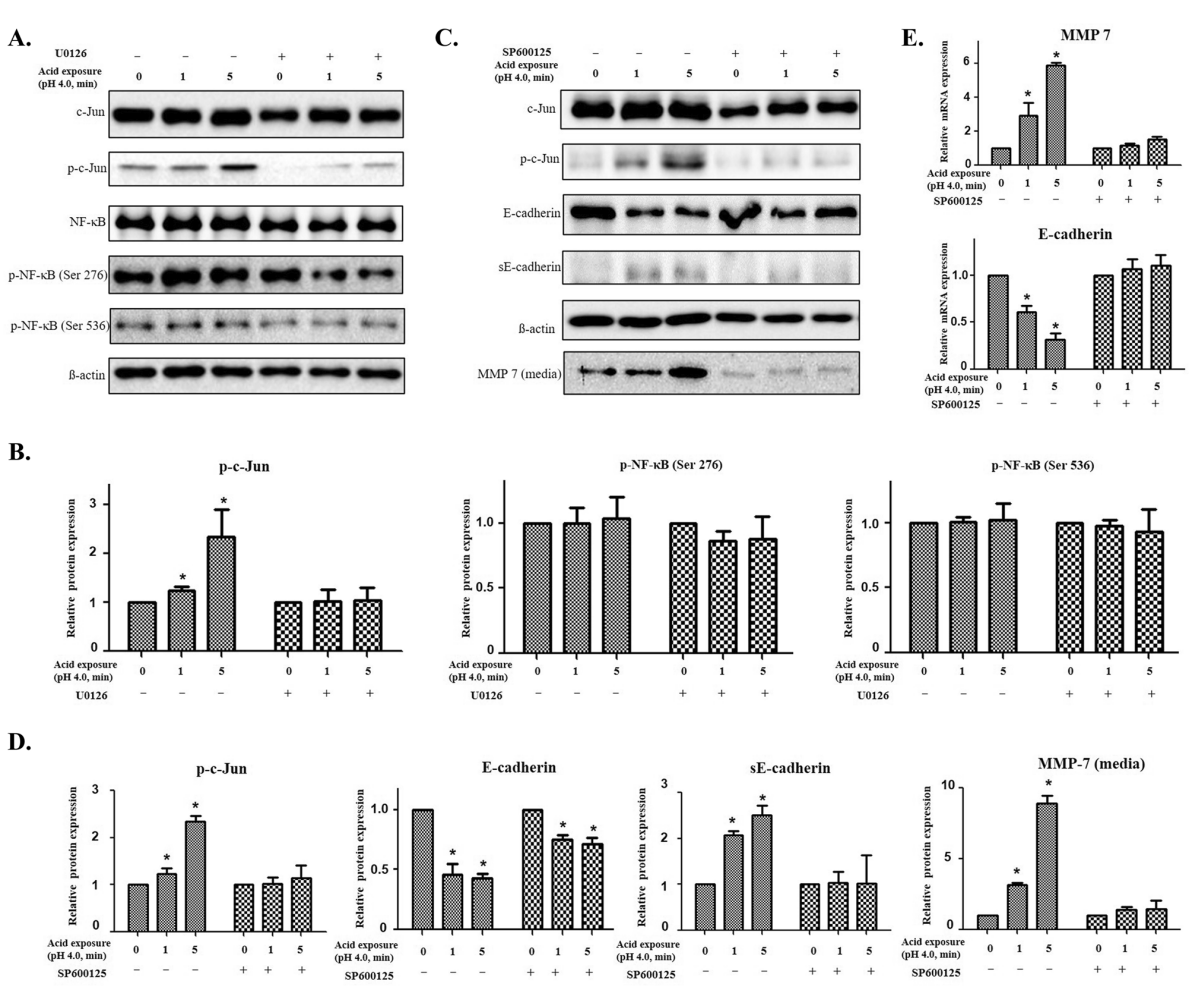
Expression of transcription factors influenced by ERK inhibition. **A** The phosphorylation of c-Jun increased in proportion to the acid exposure time, and it was reduced by U0126. **B** The relative expressions of transcription factors based on β-actin level. **C** The treatment of SP600125 on the acid-treated cells suppressed the increase of secreted MMP-7 and elicited the recovery of E-cadherin degradation. **D** The relative expressions of phosphorylated c-Jun, E-cadherin, and soluble E-cadherin based on β-actin level. The relative expression of MMP-7 in the acid-exposed medium compared to the medium without acid exposure. **E** The treatment of SP600125 suppressed the transcriptional expression of MMP-7 that was increased by acid treatment, while facilitated the transcriptional expression of E-cadherin that was inhibited by acid treatment

To identify the transcription factor regulated by ERK1/2, the phosphorylation levels of MAPK-related transcription factors, c-Jun, a member of AP (Activator protein)-1 family, and NF-κB were examined following treatment with the ERK1/2 inhibitor. We found that the phosphorylation of c-Jun increased in a manner dependent on the acid exposure time, which was reduced following treatment with U0126 (Fig. 3A and B). However, the phosphorylation of NF-κB was not affected following acid or inhibitor treatment (Fig. 3A and B). These results indicate that c-Jun is a major transcription factor that is regulated in acid-treated pharyngeal cells. When SP600125 (c-Jun inhibitor) was used, the increase in the amount of secreted MMP-7 was alleviated. This result was accompanied by the recovery of E-cadherin degradation, suggesting that the cell–cell interaction was maintained by the suppression of MMP-7 expression through the increased c-Jun activity of the MAPK signaling pathway (Fig. 3C and D). In addition, when RT-qPCR was performed on MMP 7 and E-cadherin along with c-Jun inhibition, the increased expression of MMP-7 was suppressed, while the decrease in E-cadherin expression was recovered (Fig. 3E).

### E-cadherin cleavage occurs through the ROS/ERK/c-Jun pathway

**Fig. 4:**
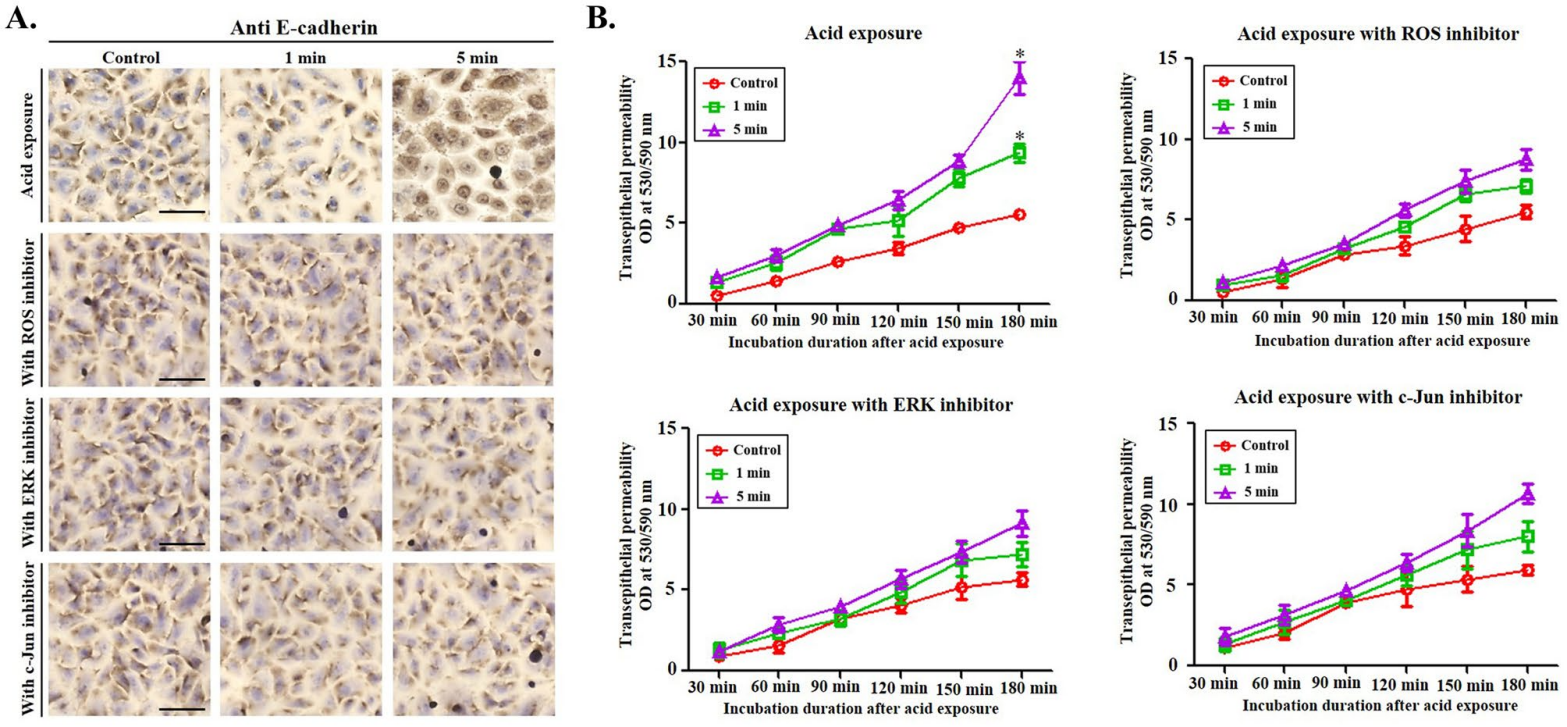
Effects of inhibitors on the changes in E-cadherin cleavage in human pharyngeal epithelial cells. **A** Depending on the acid exposure time, E-cadherin expression in the cell–cell junction decreased, which is inhibited by treatment with reactive oxygen species (ROS), extracellular signal-regulated kinase (ERK), and c-Jun inhibitors. (Bar, 200 μm). **B** Transmembrane permeability increases following acid exposure and is inhibited by treatment with ROS, ERK, and c-Jun inhibitors. (red line, control; green line, acid exposure for 1 min; purple line, acid exposure for 5 min)

To evaluate the effect of inhibition of ROS/ERK/c-Jun signaling on cell–cell interactions in an acidic environment, the pharyngeal epithelial cells were stained against E-cadherin after acid treatment, and their transepithelial permeability was measured. Depending on the acid exposure time, the amount of stained E-cadherin between the cells decreased (Fig. 4A) and transepithelial permeability increased (Fig. 4B). When the acid-exposed pharyngeal epithelial cells were treated with the ROS, ERK, or c-Jun inhibitor, the reduced amount of intracellular E-cadherin was recovered (Fig. 4A), and the increase in transepithelial permeability was reduced (Fig. 4B). Interestingly, treatment with the ROS inhibitor reduced transepithelial permeability to a greater extent than treatment with the ERK or c-Jun inhibitor (Fig. 4B). Therefore, ROS seems to have the potential to disrupt cell–cell interactions through various mechanisms apart from the ERK/c-Jun signaling pathway.

## Discussion

LPRD, with a high prevalence rate, exhibits some typical symptoms, such as globus sensation and throat pain, which are associated with pharyngeal mucosal damage. Although pharyngeal damage is caused by the reflux of gastric contents, including gastric acid, pepsin, and bile juice, there is controversy over which specific gastric content plays a key role in the occurrence of LPRD [19]. Furthermore, although recent studies have reported the in vitro models of LPRD [20], only a few have focused on the mechanisms underlying the pathogenesis of this disease.

The present study demonstrated that the exposure of human pharyngeal epithelial cells to acid increases the generation of ROS in a manner dependent on the time of exposure, and only the ERK signaling pathway was significantly activated among all the MAPK signaling pathways regulated by ROS. In addition, the levels of c-Jun, an ERK-related transcription factor, increased in a manner dependent on the acid exposure time. All these changes caused by acid exposure increased the expression of MMP-7 and elicited the degradation of E-cadherin. By contrast, treatment with the inhibitors of ROS, ERK, and c-Jun suppressed the expression of MMP-7 and degradation of E-cadherin. Thus, the findings of the present study show that acid exposure activates the ERK1/2 signaling pathways in epithelial cells via ROS production, which leads to the synthesis of MMP-7 through the transactivation of c-Jun. Subsequently, MMP-7 causes the degradation of E-cadherin.

The known causes of LPRD include the nature of microbiome and increase in the levels or reflux of gastric acid (HCl), pepsin, and bile juice. Since the mechanisms through which each gastric content affects the occurrence of LPRD differ, the cause of the disease may vary depending on the specific reflux content and its mechanism of action. However, the common phenomenon exhibited by these four contents is that they release ROS [21]. In the current study, using an in vitro model of LPRD, we found that acid exposure significantly increased the expression of ROS and MMP, which was reduced following treatment with the antioxidant, NAC, an ROS inhibitor. Notably, a marked change in MMP-7 expression in the culture media was observed after acid and NAC treatments. This suggests that increase in the secretion of MMP-7 into the medium may be more important for the cleavage of E-cadherin than the intracellular expression of MMP-7, as most MMPs become active after secretion [22].

The MAPK signaling pathway is primarily regulated by ROS production [23]. ROSs play important roles as signaling molecules that regulate many genes, including *MMP*, and they are also involved in the regulation of MMP expression through the indirect activation of MAPK family members. In addition, it has been demonstrated that induced cell stress activates the phosphoinositide-3-kinase, ERK1/2, and p38 signaling pathways, which lead to the synthesis of MMP-7 through the transactivation of NF-κB and c-Jun in cancer cells [24]. Similarly, our study showed that p-ERK1/2 levels increased in a manner dependent on the acid exposure time, but they were suppressed following ROS inhibitor treatment, and treatment with the ERK inhibitor decreased the amount of MMP-7 secreted into the media. Collectively, these results indicate that ROS levels increase in an acidic environment, and ROSs activate ERK, increasing MMP-7 levels and leading to pharyngeal damage via E-cadherin cleavage.

Many previous studies have reported that MMP expression is regulated at the transcriptional level [25–28]. The MMP promoters harbor several *Cis* elements that regulate gene expression through various trans-activators, including AP-1/2, polyomavirus enhancer activator 3, specificity protein-1, and NF-κB. Since these transcription factors are cell- or tissue-specific, their mode of activation differs according to their response to specific stimuli or diseases [29]. In the present study, MMP-7 expression was regulated by p–c-Jun, an AP-1 transcription factor, in the pharyngeal epithelial cells exposed to an acidic environment. Accordingly, ERK1/2 activated by ROS modulated c-Jun expression among all the AP-1 transcription factors, leading to the regulation of the transcription of MMP-7. Similarly, in a previous study, it was observed that MMP-7 was expressed through the activation of the AP-1 pathway, when the cultured cells were treated with H_2_O_2_ [26, 28].

Interestingly, the decrease in transepithelial permeability was greater following treatment with the ROS inhibitor than with the ERK or c-Jun inhibitor. These results indicate that in addition to the ERK/c-Jun pathway, other mechanisms that influence the cleavage of E-cadherin by ROS may exist; however, the main mechanism involves the ROS/ERK/c-Jun pathway.

## Conclusions

The acid exposure–induced cleavage of E-cadherin in pharyngeal epithelial cells is caused by MMP-7 activation; its levels rise with the increase in ROS- and ERK1/2-mediated activation of MAPKs and enhanced phosphorylation of the transcription factor c-Jun. Even though this mechanism may be one of several mechanisms to elicit LPRD, the discovery of that will help guide future studies on LPRD. Furthermore, we believe that blocking the mechanism underlying the onset of this disease may serve as a new strategy for the treatment of LPRD.

## Supplementary Information

Below is the link to the electronic supplementary material.

Supplementary file1 (DOCX 384 KB)

## Data Availability

Not applicable.

## Abbreviations

AP: Activating protein
BMEM: Bronchial epithelial cell growth basal medium
DMEM: Dulbecco’s modified Eagle’s medium
E-cadherin: Epithelial-cadherin
ERK: Extracellular signal-regulated kinase
JNK: C-Jun N-terminal kinase
LPRD: Laryngopharyngeal reflux disease
MAPK: Mitogen-activated protein kinase
MMP-7: Matrix metalloproteinase-7
NAC: N-acetyl cysteine
NF-κB: Nuclear factor-κB
PBS: Phosphate-buffered saline
ROS: Reactive oxygen species
RT-qPCR: Reverse transcription-quantitative polymerase chain reaction
