# Correction to: Matrix metalloproteinase‑7 induces E‑cadherin cleavage in acid‑exposed primary human pharyngeal epithelial cells via the ROS/ERK/c‑Jun pathway

**DOI:** 10.1007/s00109-022-02212-4

**Published:** 2022-05-26

**Authors:** Nu‑Ri Im, Byoungjae Kim, Kwang‑Yoon Jung, Seung‑Kuk Baek

**Affiliations:** 1 Department of Otorhinolaryngology‑Head and Neck, Surgery, College of Medicine, Anam Hospital, Korea, University, 73, Inchon‑ro, Seongbuk‑gu, Seoul, South Korea; 2 College of Medicine, Neuroscience Research Institute, Korea University, Seoul, Republic of Korea

## Correction to: Journal of Molecular Medicine (2022) 100:313–322 https://doi.org/10.1007/s00109-021-02166-z

The image of Figs. 1 and 3 were interchanged in the Original published proof.

The Original article has been corrected.

