# Supplementary material for: Matrix metalloproteinase-7 induces E-cadherin cleavage in acid-exposed primary human pharyngeal epithelial cells via the ROS/ERK/c-Jun pathway
Source: J Mol Med (Berl). 2022 Jan 1;100(2):313–22. doi: 10.1007/s00109-021-02166-z (PMC8770433; doi:10.1007/s00109-021-02166-z)
Supplement: Supplementary file 1 — Supplementary file1 (DOCX 384 KB) [file 109_2021_2166_MOESM1_ESM.docx]

**Matrix metalloproteinase-7 induces E-cadherin cleavage in acid-exposed primary human pharyngeal epithelial cells via the ROS/ERK/c-Jun pathway**

**Nu-Ri Im^1^, Byoungjae Kim^1,2^, Kwang-Yoon Jung^1^, Seung-Kuk Baek^1^**

^1^Department of Otorhinolaryngology-Head and Neck Surgery, Korea University, College of Medicine, Seoul, South Korea;

^2^Neuroscience Research Institute, Korea University, College of Medicine, Seoul, Republic of Korea

**Supplementary Figure 1.** Reactive oxygen species (ROS) and matrix metalloproteinase-7 (MMP-7) levels in human pharyngeal mucosal epithelial cells exposed to acidic media. **(Full-length blots/gels of Figure 1)**

**
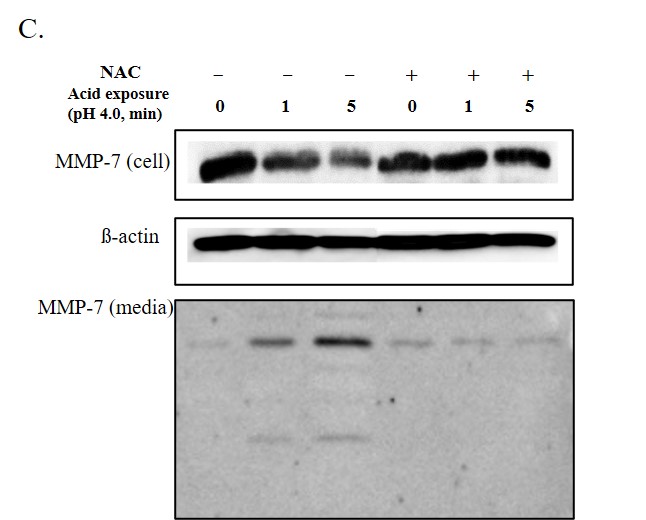
**

**Supplementary Figure 2.** Regulation of the mitogen-activated protein kinase (MAPK) pathway after acid treatment. **(Full-length blots/gels of Figure 2)**

**
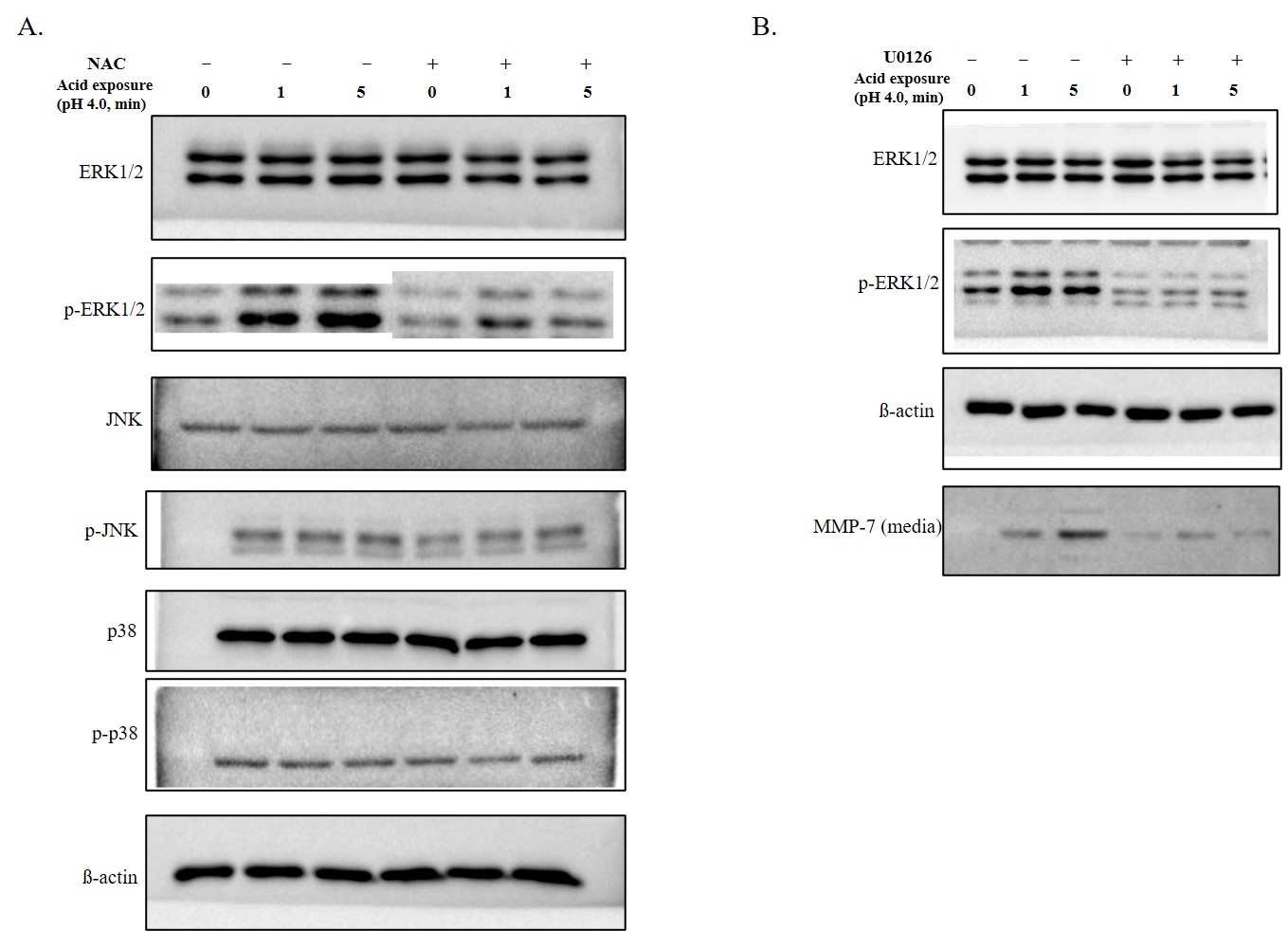
**

**Supplementary Figure 3.** Expression of transcription factors influenced by ERK inhibition. **(Full-length blots/gels of Figure 3)**

**
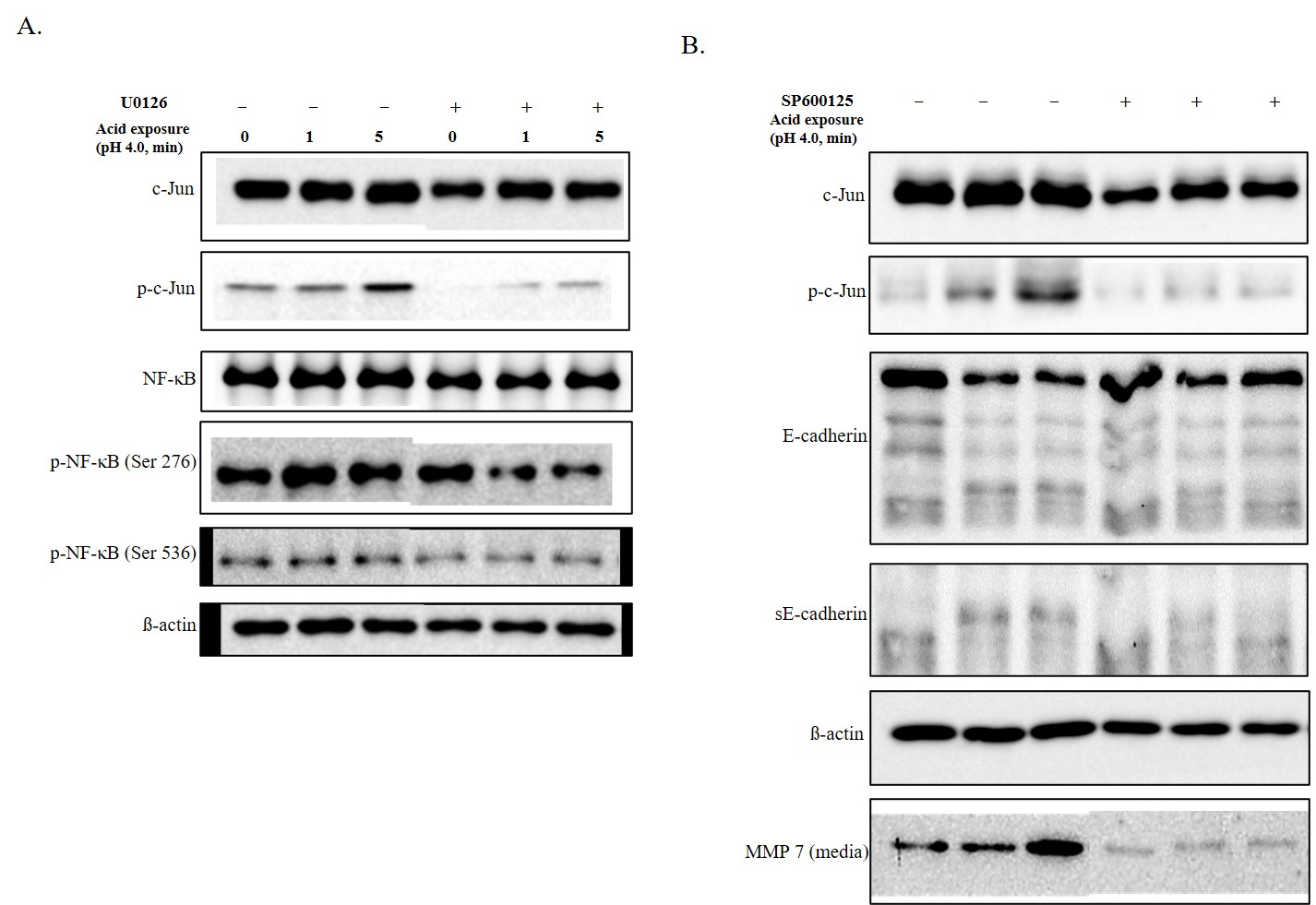
**
